# Photostability Testing of a Third-Generation Retinoid—Tazarotene in the Presence of UV Absorbers

**DOI:** 10.3390/pharmaceutics12090899

**Published:** 2020-09-22

**Authors:** Agata Kryczyk-Poprawa, István Zupkó, Péter Bérdi, Paweł Żmudzki, Justyna Popiół, Bożena Muszyńska, Włodzimierz Opoka

**Affiliations:** 1 Department of Inorganic and Analytical Chemistry, Jagiellonian University Medical College, 30-688 Kraków, Poland; wlodzimierz.opoka@uj.edu.pl; 2 Department of Pharmacodynamics and Biopharmacy, University of Szeged, 6720 Szeged, Hungary; zupko@pharm.u-szeged.hu (I.Z.); berdi.peter@pharm.u-szeged.hu (P.B.); 3 Interdisciplinary Center for Natural Products, University of Szeged, 6720 Szeged, Hungary; 4 Department of Medicinal Chemistry, Jagiellonian University Medical College, 30-688 Kraków, Poland; pawel.zmudzki@uj.edu.pl; 5 Department of Pharmaceutical Biochemistry, Jagiellonian University Medical College, 30-688 Kraków, Poland; justyna.popiol@uj.edu.pl; 6 Department of Pharmaceutical Botany, Jagiellonian University Collegium Medicum, 30-688 Kraków, Poland; muchon@poczta.fm

**Keywords:** tazarotene, photostability studies, photocatalytic degradation, benzophenones, TiO_2_, ZnO

## Abstract

Exposure of a drug to UV irradiation could affect its physicochemical properties. Hence, photostability testing is essential for topically administered drugs. Tazarotene, a receptor-selective, third-generation retinoid, is commonly used to treat acne vulgaris and psoriasis. In the present study, an in-depth analysis of the photostability of tazarotene in ethanolic solution in the presence of zinc oxide and/or titanium dioxide as well as benzophenone-type UV filters was performed. Eleven presumed products were derived from the photocatalytic degradation of tazarotene using ultra-performance liquid chromatography–tandem mass spectrometry, and transformation pathways were proposed. The degradation process mainly affected the 4,4-dimethyl-3,4-dihydro-2*H*-thiopyran moiety. The fragments most susceptible to oxidation were the methyl groups and the sulfur atom. Moreover, in the presence of sulisobenzone, under UV irradiation, tazarotene was subjected to a degradation process, which resulted in two photodecomposition products. *In silico* studies performed by OSIRIS Property Explorer demonstrated that five of the degradation products could be harmful in terms of the reproductive effects, which are associated with 3,4-dihydro-6-methyl-2*H*-1-benzothiopyran 1,1-dioxide, while one of them demonstrated potential irritant activity. The cytotoxic properties of the degradation products of tazarotene were assessed by MTT assay on a panel of human adherent cancer cells. Time- and concentration-dependent growth inhibition was evidenced in ovary (A2780) and breast (MDA-MB-231) cancer cell lines. The potential implication of the outcomes of the present research requires further studies mainly concerning the photostability of tazarotene in the topical formulations.

## 1. Introduction

Tazarotene (ethyl 6-[2-(4,4-dimethyl-2,3-dihydrothiochromen-6-yl)ethynyl]pyridine-3-carboxylate) is a third-generation topical retinoid, which is applied to the skin. It is a prodrug, which undergoes in vivo deesterification in its active form—the cognate carboxylic acid of tazarotene [1]. Tazarotene acid selectively binds to the retinoic acid receptors—RAR-β and RAR-γ [2,3]. This drug is used in the treatment of mild-to-moderate facial acne vulgaris and plaque psoriasis affecting nearly 20% of the body surface [4,5,6]. Acne vulgaris is a common, chronic skin disease, which affects approximately 90% of humans across the world at some point in their lives. Topical retinoids are currently used in the first-line treatment of the disease. Furthermore, tazarotene is used to treat other skin conditions such as photoaging and facial mottled hyper- and hypopigmentation [7]. It slightly penetrates across the skin, and 10 h after its application, ~2% of the dose reaches the viable epidermis and dermis [8]. Tazarotene gel is also used with narrow-band UVB phototherapy for psoriasis because it enhances the efficiency of the therapy and enables a significant reduction of the irradiation dose [9,10]. Like other topical retinoids, tazarotene could cause photosensitivity reactions—generally mild and reversible phototoxic or photoallergic reactions—and display minor photoirritant potential [1,11,12].

Besides, topical photoprotection is an extremely important area of dermatologic research on skin cancer prevention strategies [13]. A better understanding of the potential interactions between different substances applied simultaneously to the skin, e.g. UV absorbers and active pharmaceutical ingredients, is essential for ensuring the safe and effective administration of drug products [14]. The behavior of these substances is not predictable from independent photostability testing [15,16]. Hence, it is imperative to evaluate the combinations of substances used in the formulations as well as active pharmaceutical ingredients used with cosmetic ingredients [17,18]. The previous investigation of the photostability of terbinafine, in both solutions and formulations in the presence of UV absorbers such as TiO_2_, ZnO, avobenzone, 3-(4-methylbenzylidene)camphor, octocrylene, benzophenone-1, and benzophenone-2, showed that UV absorbers had a diverse impact on terbinafine stability [19]. It should be emphasized that photostability testing is an integral part of the stability studies included in the Q1A–Q1F Quality Guidelines of The International Council for Harmonization of Technical Requirements for Pharmaceuticals for Human Use (ICH). The guideline concerning the Photostability Testing of New Drug Substances and Products—Q1B was implemented in Europe on 1 December 1996 and in the USA on 1 May 1997 [20]. The photoinstability of a drug could impact its effectiveness and lead to the formation of products with unknown activity [21]. The lack of detailed information about the photostability of tazarotene prompted us to perform in-depth research on this third-generation retinoid.

Tazarotene is considered to be photostable; however, it is specifically used by young people who spend a lot of time outside and are exposed to UV irradiation, which increases the frequency of the simultaneous use of UV filters. Hence, there is an urgent need to study the interactions between tazarotene and UV absorbers under UV irradiation. The present study aimed to assess the impact of selected UV absorbers extensively used in sunscreens and cosmetics on the photostability of tazarotene in ethanol solutions under UV irradiation. Benzophenone compounds were chosen due to their widespread employment in cosmetics, photostability, and wide-ranging spectrum protection against not only UVB (290–320 nm) in particular but also UVA (320–400 nm) [22,23]. Out of the twelve benzophenone-type UV absorbers, the following four were selected for this study: benzophenone-1, benzophenone-2, benzophenone-3 (oxybenzone), and benzophenone-4 (sulisobenzone). Additionally, avobenzone, which is one of the most common UVA filters, was investigated. Due to its rapid photodegradation, avobenzone should be included with a stabilization agent—such as octocrylene [24]. Furthermore, an assessment of the effect of TiO_2_ and ZnO nanoparticles, which could be used as physical UV filters in sunscreens, on the stability of tazarotene under UV irradiation was performed. According to the regulations of the US Food and Drug Administration (FDA), ZnO and TiO_2_ are used in sun protection products in maximum concentration up to 25% [25]. It is universally accepted that ZnO and TiO_2_ as semiconductors could be used in heterogeneous photocatalysis [26,27]. Both the oxides display high photocatalytic activity and are chemically and photochemically stable. Nevertheless, there is very little information in the research literature about the potential photocatalytic degradation products of tazarotene. The overarching goal of the conducted in vitro tests was to determine the plausible structures of the photoproducts formed under experimental conditions and to evaluate their cytotoxicity.

## 2. Materials and Methods

### 2.1. Reagents

Tazarotene (ethyl 6-[2-(4,4-dimethyl-2,3-dihydrothiochromen-6-yl)ethynyl]pyridine-3-carboxylate) was purchased from Sigma-Aldrich (St. Louis, MO, USA). High-performance liquid chromatography (HPLC)-grade methanol, acetonitrile, and formic acid (98%) were purchased from J.T. Baker (Phillipsburg, NJ, USA). Ethanol absolute for HPLC ≥99.8% was obtained from POCH (Gliwice, Poland). Water (quadruple-distilled) with a conductivity of less than 1 μS cm^−1^ was prepared using the S2-97A2 distillation apparatus (ChemLand, Stargard Szczecin, Poland). Zinc oxide (nanopowder <100 nm particle size) and titanium (IV) oxide (anatase, nanopowder < 25 nm particle size, 99.7% trace metals basis) were purchased from Sigma-Aldrich (St. Louis, MO, USA). The UV absorbers—benzophenone-1, benzophenone-2, benzophenone-3, benzophenone-4, avobenzone, and octocrylene—were obtained from Sigma-Aldrich (St. Louis, MO, USA).

### 2.2. Reagent Solutions Preparation

The stock solution of tazarotene was prepared at a concentration of 0.2 mg mL^−1^ in a 50-mL volumetric flask by dissolving the weighted portion of the standard substance in ethanol and was then stored at 2–8 °C for no longer than three days. For the method validation, six solutions with tazarotene concentrations of 0.01, 0.025, 0.05, 0.1, 0.15, and 0.2 mg mL^−1^ were prepared. For the assessment of photocatalytic degradation, suspensions of TiO_2_, ZnO, or TiO_2_/ZnO (1:1, *w/w*) in ethanol were prepared before each experiment by adding 100 mg of oxide to 5 mL of ethanol and were then sonicated in an ultrasonic bath for 3 min. The solutions of organic UV absorbers were made at a concentration of 1 mg mL^−1^.

### 2.3. Photostability Assessments

The investigated solutions were placed in quartz Petri dishes with Ø 50 × 12 mm (Hornik, Poznań, Poland), which were protected against evaporation with parafilm. The irradiation experiments were carried out in a solar light simulator (Suntest CPS+, Atlas, Germany) which included an optical filter cutting off wavelengths shorter than 290 nm and an infrared-block filter to minimize the thermal effects. The samples were irradiated for 1 h at 500 W m^−2^ (cumulative dose of UV radiation 218 kJ m^−2^). For photostability analyses of tazarotene in ethanol, 2 mL of the investigated solution at a final concentration of 0.1 mg mL^−1^ was irradiated.

Photocatalytic degradation tests were performed by adding 1 mL of tazarotene solution, 0.5 mL of one of the prepared suspensions of TiO_2_, ZnO, or TiO_2_/ZnO along with the right amount of ethanol to achieve the final concentration of tazarotene. After the stipulated time of irradiation, the quartz dishes were withdrawn, and the samples were filtered through 0.45 µm syringe filters before the ultra-performance liquid chromatography–tandem mass spectrometry (UPLC–MS/MS) analysis. Analogously prepared samples, which were protected with aluminum foil before irradiation, were used as the dark control. For UV absorbers, analogously prepared solutions containing 1 mL of 0.2 mg mL^−1^ tazarotene solution, 0.8 mL of ethanol, and 0.2 mL of benzophenone-type UV absorber solution in ethanol or 0.1 mL of avobenzone +0.1 mL of octocrylene were exposed to UV irradiation in a solar light simulator.

### 2.4. In Vitro Cytotoxicity Assays

#### 2.4.1. Investigated Solutions

The stock solution of tazarotene in ethanol was prepared in a 10-mL flask at a concentration of 10 mM. The prepared samples containing 2 mL of the above solution with the additional 10 mg of TiO_2_/ZnO (1:1, *w/w*) were placed into quartz dishes. The samples were irradiated for 0.5, 1, and 2 h in a solar light simulator. Later, the samples were filtered through a 0.45 μm filter and subjected to cytotoxicity testing.

#### 2.4.2. Cell Lines and Cell Growth Inhibitory Assay

Human cancer cell lines isolated from breast (MDA-MB-231), cervix (HeLa), and ovary (A2780) cancers were purchased from European Collection of Cell Cultures (ECCAC, Salisbury, UK). Cells were cultivated in minimal essential medium supplemented with 10% fetal bovine serum, 1% antibiotic–antimycotic mixture, and nonessential amino acids. All media and supplements were obtained from Lonza Group Ltd. (Basel, Switzerland). Near-confluent cells were seeded onto a 96-well microplate at a density of 5000 cells/well, and after overnight standing, new medium containing the tested substance was added. After incubation for 72 h at 37 °C in humidified air containing 5% CO_2_, the viability of the cells was determined by the addition of 20 μL of MTT (3-(4,5-dimethylthiazol-2-yl)-2,5-diphenyltetrazolium bromide, 5 mg mL^−1^) solution. After a 4h contact period, the medium was removed, and the precipitated formazan crystals were dissolved in 100 μL of dimethyl sulfoxide (DMSO) during a 60 min period of shaking. Finally, the produced formazan was assayed at 545 nm, using a microplate reader utilizing wells with untreated cells as control [28]. All in vitro experiments were carried out on two microplates with at least five parallel wells.

### 2.5. In Silico Toxicity Prediction

OSIRIS Property Explorer was used to predict mutagenicity, tumorigenicity, irritation, and reproductive effects of tazarotene and its eleven photocatalytic degradation products [29]. The investigated structures were used to predict the toxicity risks related to the specific risk category.

### 2.6. Ultra-Performance Liquid Chromatography–Tandem Mass Spectrometry Analysis

The UPLC–MS/MS system equipped with a Waters ACQUITY^®^ UPLC^®^ (Waters Corporation, Milford, MA, USA) and a Waters TQD mass spectrometer (electrospray ionization (ESI) mode tandem quadrupole) was used for the analysis. The investigated compound was analyzed on a Acquity UPLC BEH (bridged ethyl hybrid) C18 column (2.1 × 100 mm, 1.7 µm), comprising an Acquity UPLC BEH C18 VanGuard precolumn (2.1 × 5 mm, 1.7 µm) eluted under gradient conditions using 95% to 0% of eluent A over 10 min, at a flow rate of 0.3 mL min^−1^. The column temperature was maintained at 40 °C. Eluent A was water/formic acid (0.1%, *v/v*), while eluent B was acetonitrile/formic acid (0.1%, *v/v*). Chromatograms were acquired using a Waters eλ PDA detector. The concentration (%i) of tazarotene after its degradation was calculated as the quotient of the peak area (Ai) to the sum of all peak areas (∑A) on chromatograms according to the formula, %i = (Ai/(∑A) × 100). In the case of organic UV absorbers, the individual peak areas of the analyzed UV filters were not included in the total peak area. Chromatograms were recorded using the Waters eλ PDA detector. The spectra were analyzed at 200–700 nm with 1.2 nm resolution and a sampling rate of 20 points s^−1^. MS detection settings of Waters TQD mass spectrometer were as follows: source temperature—150 °C, desolvation temperature—350 °C, desolvation gas flow rate—600 L h^−1^, cone gas flow—100 L h^−1^, capillary potential—3.00 kV, and cone potential—30 V. Nitrogen was used for both nebulizing and drying gases. The optimum collision energy was determined to be 50 eV. The ion spectra were obtained by scanning from 50 to 350 *m/z*. MassLynx V 4.1 (Waters) software was used for data acquisition.

### 2.7. Statistical Analysis

Statistical analyses were performed using Statistica v. 12 (StatSoft) and GraphPad Prism (GraphPad 6 Software, San Diego, CA, USA). For analysis of cell death, two-way analysis of variance with Tukey’s multiple comparisons test was carried out. Differences were considered statistically significant in comparison with untreated controls at *p* ≤ 0.05.

## 3. Results and Discussion

### 3.1. Method Validation 

**Figure 1:**
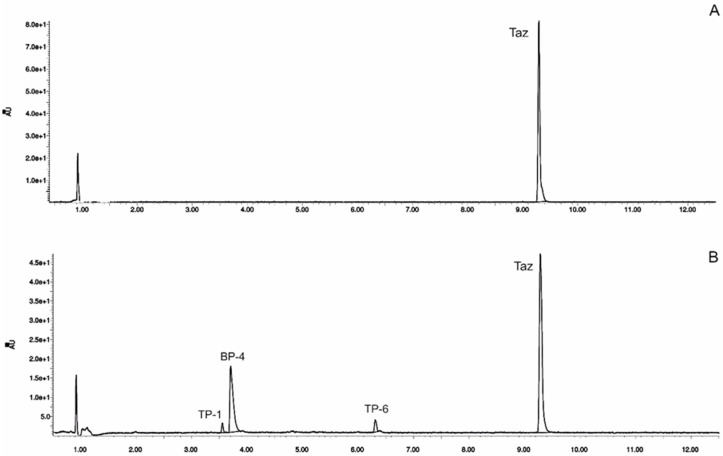

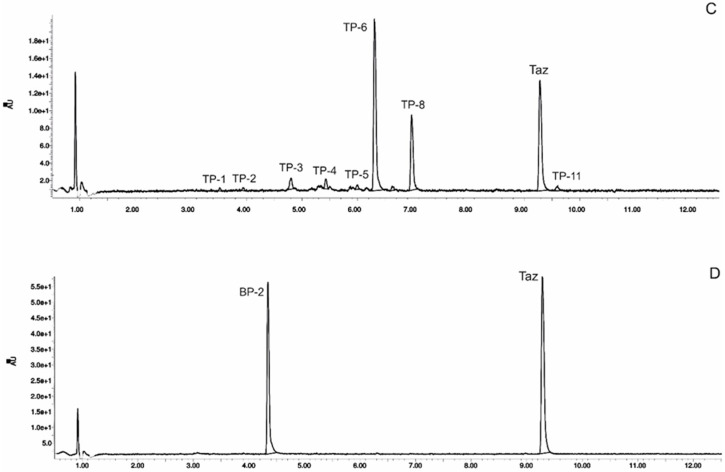
High-performance liquid chromatography chromatograms of tazarotene (TAZ) following UV irradiation in the presence of selected UV absorbers: (**A**) tazarotene standard, (**B**) tazarotene with sulisobenzone (BP-4), (**C**) tazarotene with TiO_2_ and ZnO (TP- degradation products of tazarotene), (**D**) tazarotene with benzophenone-2 (BP-2).

The main objective of applying the UPLC method was to confirm its applicability for the assessment of tazarotene in the presence of its degradation products. The developed method was suitable for determining tazarotene and its degradation products in the tested ethanolic solutions. The specificity of the method was evaluated through the assessment of blank chromatograms, chromatograms of the pure standard substance, and chromatograms of tazarotene after degradation. Furthermore, the resolution of the investigated benzophenone-type UV filters, avobenzone, octocrylene, and tazarotene was verified. The chromatographic separation of the other selected UV filters and tazarotene was satisfactory, both before and after UV irradiation (Figure 1). The results were validated for linearity, precision, and accuracy [15]. Least squares linear regression was performed to statistically evaluate the relationship between the peak areas and the concentration of bexarotene. The linearity was determined for the concentration range from 0.01 to 0.2 mg mL^−1^, with a correlation coefficient and a determination coefficient (R^2^) of 0.9991 and 0.9982, respectively. The Shapiro–Wilk test was used to validate the normality assumption; the significance value (*p* = 0.17151) was greater than 0.05, and hence, the residuals were considered to be normally distributed. The precision determined from the analysis of the six replicates of tazarotene solutions at a single concentration of 0.1 mg mL^−1^ was lower than 1.29% (%RSD), and the recovery rate at three concentration levels (80%, 100%, and 120%) ranged from 97.5% to 101.90%.

### 3.2. Identification of Photocatalytic Degradation Products of Tazarotene

The impact of UV irradiation on tazarotene photostability in the presence of TiO_2_ and/or ZnO nanoparticles was assessed. The photocatalytic degradation products of tazarotene were identified with the help of the UPLC–MS/MS analysis and supported with fragmentation patterns obtained from MS/MS experiments. Table 1 presents the proposed structures of the degradation products, and Table 2 depicts the proposed fragmentation patterns of tazarotene and its degradation products. It was found that the degradation process mainly affected the 4,4-dimethyl-3,4-dihydro-2*H*-thiopyran moiety. The fragments most susceptible to oxidation were the methyl groups and the sulfur atom. The oxidation of methyl groups led to the degradation products—TP-1–TP-5, TP-7, TP-9, and TP-10. The oxidation of the sulfur atom to sulfoxide and sulfone moiety was observed for the degradation products TP-1–TP-8, ultimately resulting in the elimination of the sulfur-containing moiety, as observed for products TP-9 and TP-10. The elimination of the ester moiety was observed only for the minor product—TP-11. TP-6 and TP-8 were the earliest appearing products and dominant in terms of quantity. TP-6 has also been regarded as a hypothetical product of peroxide oxidation or photolytic exposure of tazarotene [30]. In 2020, a publication about the degradation chemistry of tazarotene was published, in which eleven degradation products of the investigated drug were characterized [31]. Forced degradation studies included the photolytic degradation of tazarotene in the following solutions: 0.1 M HCl, 0.1 M NaOH, or CH_3_CN:H_2_O (80:20, *v/v*), and tazarotene in the solid state. TP-6 was one of the presumed products of photolysis in acid and neutral conditions. The oxidative degradation of tazarotene performed in 3% H_2_O_2_ at room temperature gave rise to two products with the mass of protonated forms—*m/z* 368.1308 and 384.1258, corresponding to TP-6 and TP-8, respectively [31]. Analysis of the synthesis and degradation process of tazarotene revealed four impurities, including transformation to TP-6 and TP-8 through oxidation [4]. Exposure of tazarotene to 1.2 million lux hours resulted in the degradation of 8.85% of the initial quantity with the formation—mainly Imp-B, that is TP-6 [32]. We did not identify tazarotenic acid, the active metabolite of tazarotene, which is the major degradation product formed during hydrolysis [4,26,33]. Tazarotenic acid does not accumulate in the body over time. The concentrations of tazarotenic acid in the plasma of patients with psoriasis were found to be 0.45 ± 0.78 μg L^−1^ (after 0.05% tazarotene gel) and 0.83 ± 1.22 μg L^−1^ (after 0.1% tazarotene gel) [8]. The maximum average plasma concentrations of tazarotenic acid after topical application of 0.1% of tazarotene cream on the face were less than 0.25 μg L^−1^ [34]. It is metabolized to sulfoxide and more polar compounds and eliminated from the body via urine and feces [3,8]. The binding to plasma proteins, which plays a key role in the distribution, elimination, and therapeutic effectiveness of drugs, was greater than 99% [8]. After topical administration, tazarotene and tazarotenic acid metabolize via oxidation to sulfoxides, sulfones, and other polar metabolites [8,35]. It appears that there are some similarities between the known in vivo metabolic pathways of tazarotene and those postulated for its in vitro photocatalytic degradation. In both the cases, the sulfoxide (TP-6) and sulfone (TP-8) of tazarotene were identified.

### 3.3. UV Irradiation of Tazarotene in the Presence of Organic UV Absorbers

**Figure 2:**
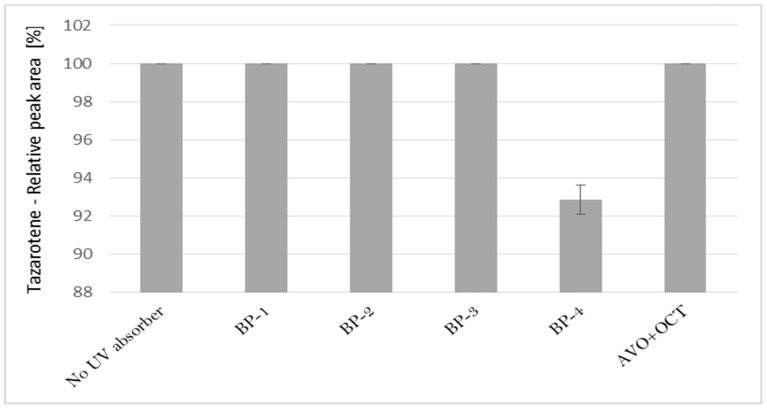
The relative peak area of tazarotene following UV irradiation in the presence of UV absorbers: BP-1 (benzophenone-1), BP-2 (benzophenone-2), BP-3 (benzophenone-3), BP-4 (benzophenone-4), and AVO + OCT (avobenzone + octocrylene).

The impact of UV irradiation on tazarotene stability in the presence of benzophenone-type UV filters was assessed. Benzophenone-1, benzophenone-2, benzophenone-3, and benzophenone-4 were selected for the study. Besides, the combination of avobenzone and octocrylene was also tested in this experiment. This is a popular combination of UV filters widely used in sunscreens. To balance the instability of avobenzone, octocrylene acts as a photostabilizer and UV absorber. Two of the investigated benzophenones—oxybenzone and sulisobenzone—with peak absorption λ_max_ equal to 286 and 324 nm have been approved by the FDA. According to the FDA regulations, the maximum recommended concentrations of benzophenone-3, benzophenone-4, avobenzone, and octocrylene are 6%, 10%, 3%, and 10%, respectively [25]. Figure 2 displays the relative peak areas of tazarotene to illustrate the impact of UV absorbers on its photostability under experimental conditions. Tazarotene was stable in all cases, excluding sulisobenzone, where a slight degradation was observed. Two additional peaks were identified on the chromatograms (Figure 1B), and two peaks in the spectrum were at *m/z* 412.18 (TP-1) and 368.18 (TP-6). The degradation process occurred through the oxidation of the sulfur atom to sulfoxide and the subsequent oxidation of the methyl groups. No photodegradation products were observed in all the dark control samples, which indicate the stability of tazarotene in the absence of UV irradiation under the applied conditions.

### 3.4. Cytotoxic Risk Assay

**Figure 3:**
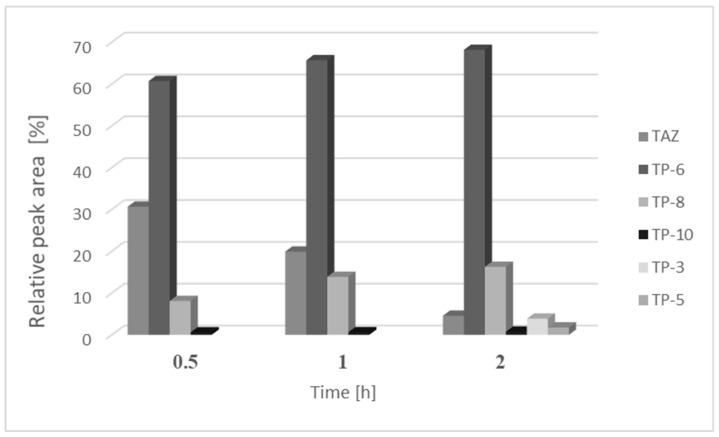
Average relative peak areas of tazarotene and its main photocatalytic degradation products identified in the tested solutions after 0.5, 1, or 2 h of UV irradiation in the presence of TiO_2_/ZnO. TAZ—tazarotene; TP-3, TP-5, TP-6, TP-8, and TP-10—photocatalytic degradation products of tazarotene.

Figure 3 depicts the relative peak area of tazarotene and its photocatalytic degradation products presented in chromatograms following 0.5, 1, and 2 h of UV irradiation of the examined solutions. The leading products present in all chromatograms were TP-6, TP-8, and TP-10. After 2 h of UV irradiation of the investigated solutions, all the identified phototransformation products of tazarotene were noted on chromatograms.

**Figure 4:**
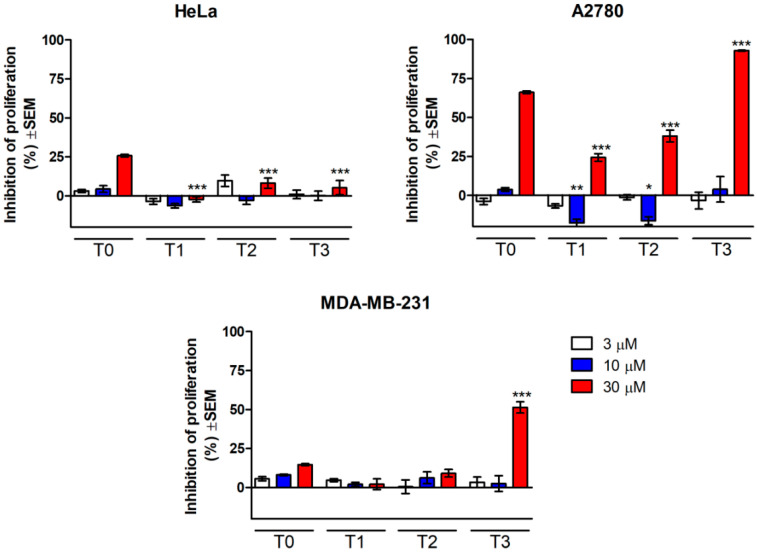
Changes in the cell viability of HeLa, A2780, and MDA-MB-231 cancer cells caused by the solutions of tazarotene following photocatalytic degradation. Cells were treated with 3, 10, and 30 μM solutions of tazarotene after UV irradiation (T0—without irradiation, T1—0.5 h of irradiation, T2—1 h of irradiation, and T3—2 h of irradiation): * *p* < 0.05, ** *p* < 0.01, and *** *p* < 0.001.

The cell growth-inhibitory activities of tazarotene following its photocatalytic degradation in experimental conditions were determined in vitro against cervical (HeLa), breast (MDA-MB-231), and ovary (A2780) cancer cell lines by means of MTT assays. Cells were treated with 3, 10, and 30 μM solution after UV irradiation (T0—without irradiation, T1—0.5 h, T2—1 h, and T3—2 h). The results are shown in Figure 4. The in vitro cytotoxicity risk evaluation based on the photocatalytic degradation of tazarotene indicated the antiproliferative action at the concentration of 30 μM. A2780 cells exhibited substantial growth inhibition by 30 μM tazarotene without irradiation followed by a biphasic action in terms of the irradiation time. Samples of lower concentrations (3 and 10 μM) resulted in a modest increase of proliferation. T3 solutions generally more effectively inhibited cell proliferation than other samples, which indicated that antiproliferative metabolites could be generated during irradiation in a time-dependent manner. The behavior of HeLa cells was unique with a concentration-dependent action of tazarotene with no irradiation which was substantially less after 0.5–2 h of irradiation.

### 3.5. In Silico Toxicity Predictions

In the next step of the study, the OSIRIS Property Explorer server was deployed to calculate the toxicities of tazarotene and all its identified photodecomposition products generated under experimental conditions. Table 3 presents the outcomes of the toxicity risk assessment. Five degradation products—TP-2, TP-3, TP-4, TP-7, and TP-8—could be harmful in terms of the reproductive effects, which is associated with 3,4-dihydro-6-methyl-2*H*-1-benzothiopyran 1,1-dioxide, while one of them—TP-10—demonstrated potential irritant activity. 

## 4. Conclusions

The presented results show the significance of the photostability studies for topical agents applied to the skin. Tazarotene, a receptor-selective, third-generation retinoid, is commonly used as monotherapy and included in combination therapy for acne vulgaris and psoriasis. This study revealed the impact of the presence of UV absorbers on the photostability of tazarotene in ethanol solution under UV irradiation. The photodecomposition of tazarotene in the presence of TiO_2_, ZnO, and sulisobenzone was observed under the applied experimental conditions. Furthermore, the presumable structures of photocatalytic degradation and photodegradation products of tazarotene were identified for the first time. The *in silico* toxicity testing of degradation products indicated their theoretical toxic reproductive effects and irritant activity. The photostability studies provided valuable information to optimize the manufacturing process as well as to ensure the safe use of medications by patients. It is worth mentioning that UV filters act as photostabilizers but could also contribute to the degradation of active pharmaceutical ingredients under UV irradiation. Therefore, further efforts should be taken to verify the potential impact of UV irradiation on different compositions of substances used in topical preparations.

## Figures and Tables

**Table 1:** Proposed structures of the degradation products of tazarotene.

Compound	RT (min)	[M+H^+^]	Fragmentation Ions	Structure
TP-1	3.52	412.1	192.1, 204.1, 310.1, 366.1, 384.1	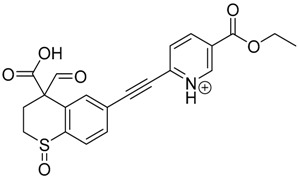
TP-2	3.94	428.1	382.1, 400.1	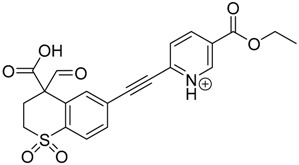
TP-3	4.80	414.1	192.1, 282.1, 296.1, 368.1, 396.1	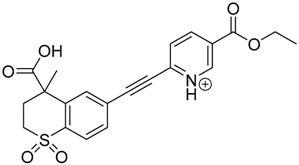
TP-4	5.43	430.1	178.1, 204.1, 294.1, 312.1, 356.1, 384.1	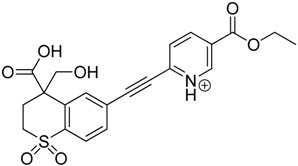
TP-5	6.00	414.1	308.0, 336.1, 350.1, 368.1, 396.1	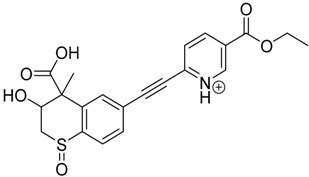
TP-6	6.30	368.1	280.1, 296.1, 308.2, 340.1	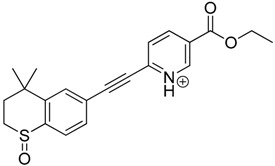
TP-7	6.62	430.1	294.1, 338.0, 366.1, 384.1, 412.1	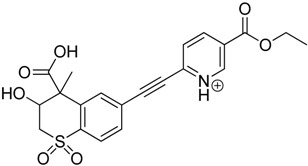
TP-8	6.97	384.1	356.1	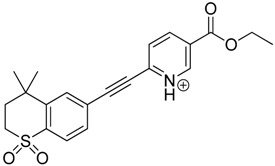
TP-9	7.47	368.1	262.1, 280.1, 269.1, 340.1	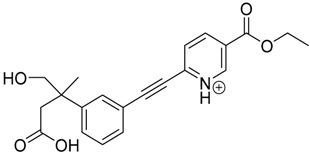
TP-10	8.49	366.1	252.1, 280.1, 294.1, 338.1	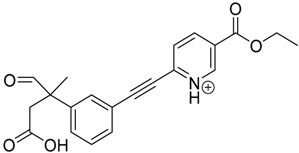
Tazarotene	9.28	352.1	222.1, 266.1, 294.1, 308.2, 324.1	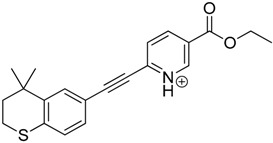
TP-11	9.60	280.1	136.1, 156.1, 236.1	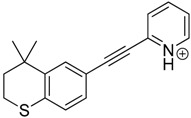

**Table 2:** Recommended fragmentation patterns.

Tazarotene
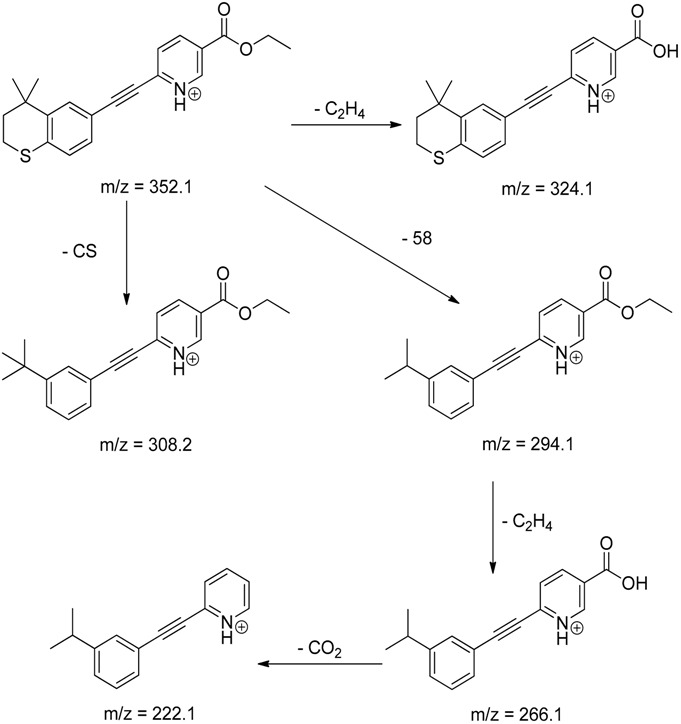
**TP-1**
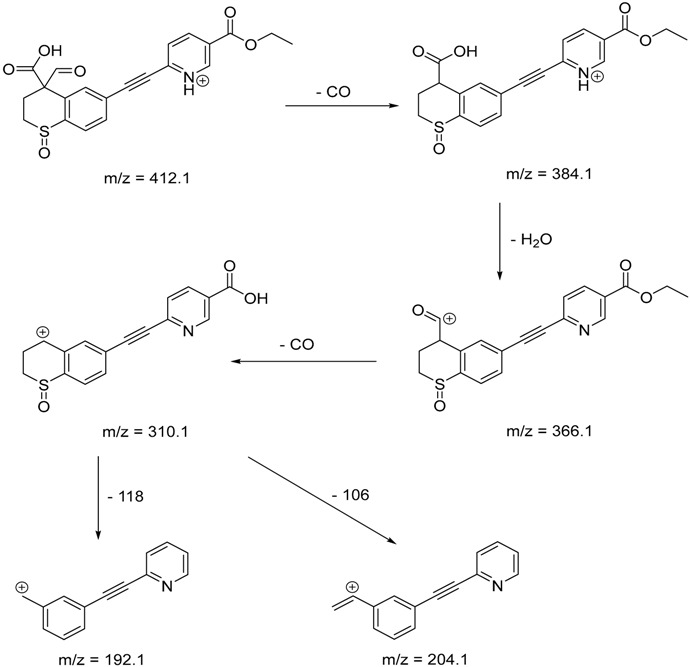
**TP-2**
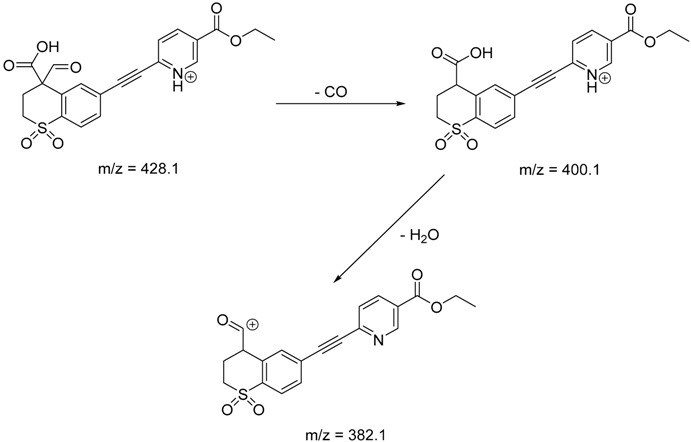
**TP-3**
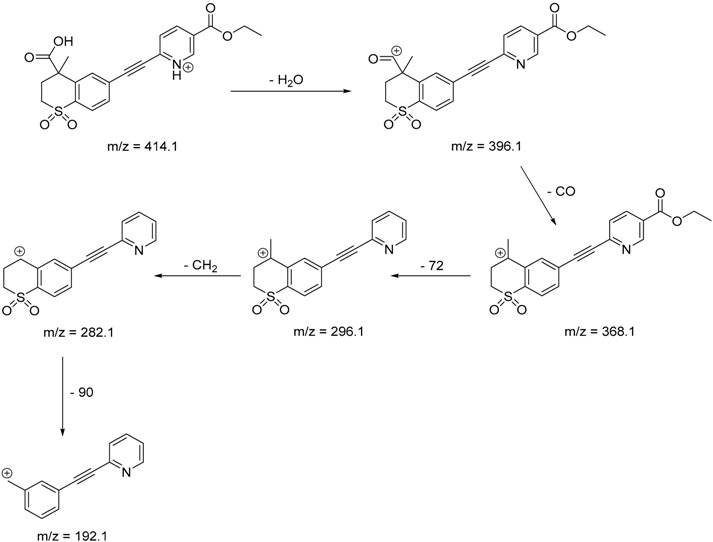
**TP-4**
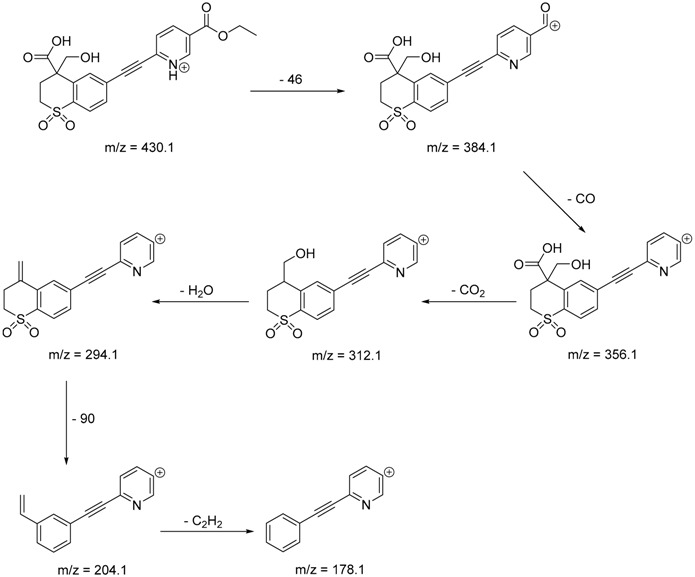
**TP-5**
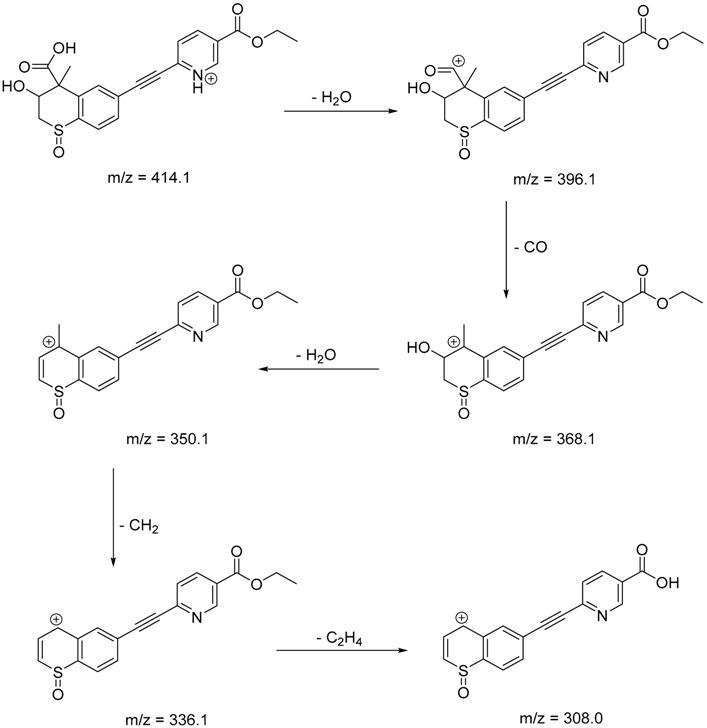
**TP-6**
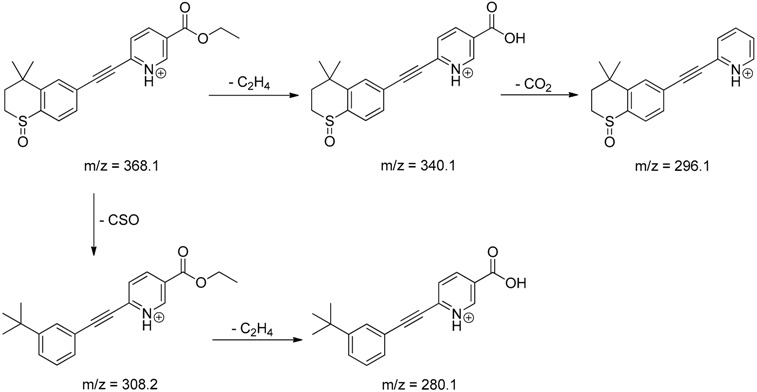
**TP-7**
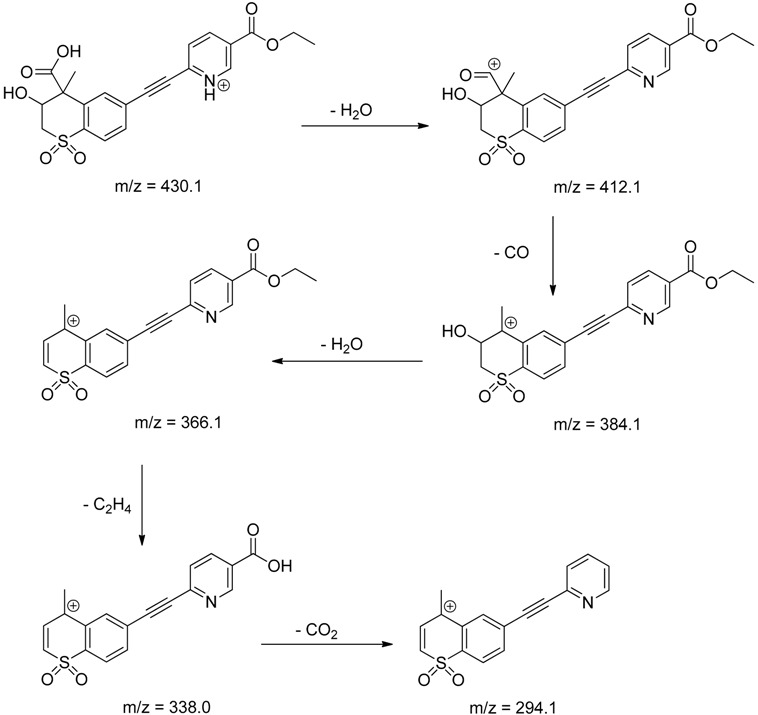
**TP-8**
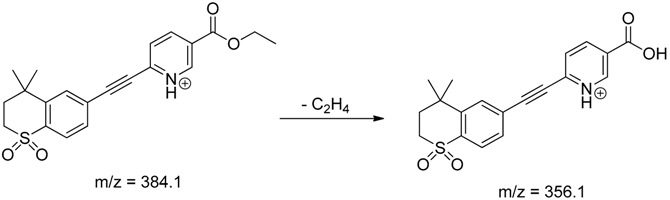
**TP-9**
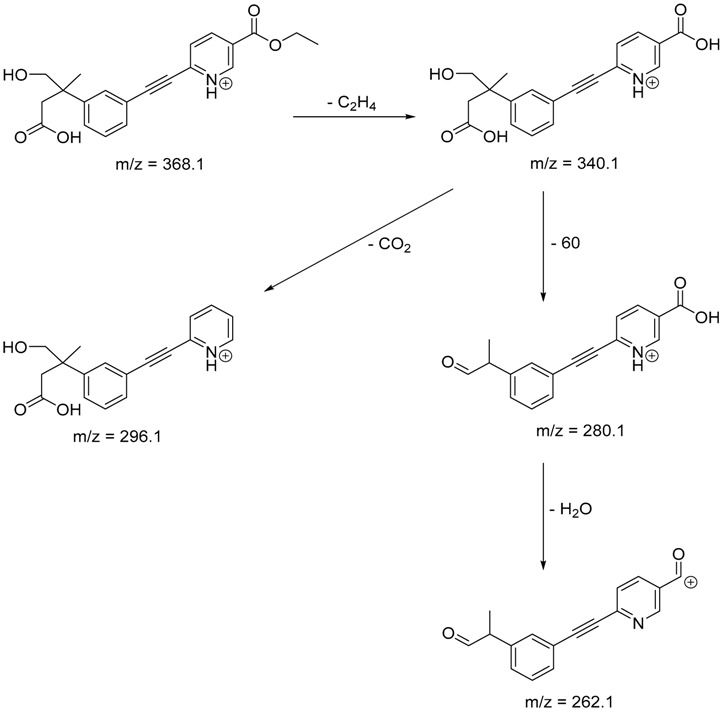
**TP-10**
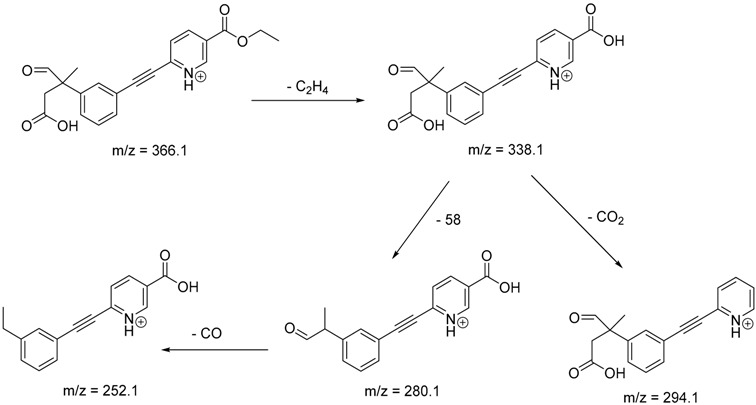
**TP-11**
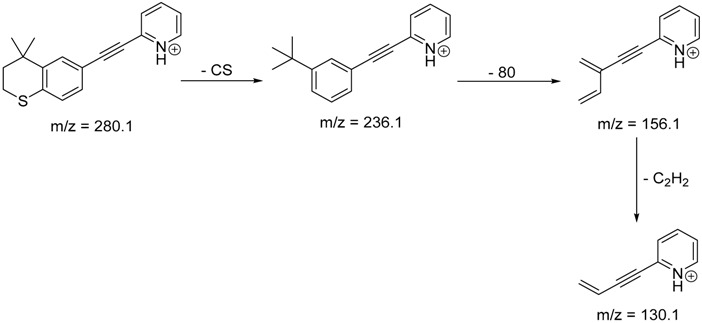

**Table 3:** Toxicity risk assessment of the photocatalytic degradation products of tazarotene (TP-1–TP-11) performed using OSIRIS Property Explorer.

Compound	Mutagenic	Tumorigenic	Irritant	Reproductive Effective
Tazarotene	-	-	-	-
TP-1	-	-	-	-
TP-2	-	-	-	+/−
TP-3	-	-	-	+/−
TP-4	-	-	-	+/−
TP-5	-	-	-	-
TP-6	-	-	-	-
TP-7	-	-	-	+/−
TP-8	-	-	-	+/−
TP-9	-	-	-	-
TP-10	-	-	+/−	-
TP-11	-	-	-	-

(-) None, (+/−) Medium.
